# A limb-girdle myopathy phenotype of *RUNX2* mutation in a patient with cleidocranial dysplasia: a case study and literature review

**DOI:** 10.1186/s12883-016-0781-2

**Published:** 2017-01-06

**Authors:** Sung-Ju Hsueh, Ni-Chung Lee, Shu-Hua Yang, Han-I Lin, Chin-Hsien Lin

**Affiliations:** 1 Department of Neurology, National Taiwan University Hospital, No. 7, Chung-Shan South Road, Taipei, 100 Taiwan; 2 Department of Medical Genetics, National Taiwan University Hospital, Taipei, 100 Taiwan; 3 Department of Orthopedics, National Taiwan University Hospital, No. 7, Chung-Shan South Road, Taipei, 100 Taiwan

**Keywords:** Cleidocranial dysplasia, RUNX2, Myopathy, Neurological system, Case report

## Abstract

**Background:**

Cleidocranial dysplasia (CCD) is a rare hereditary disorder that arises from heterozygous loss of function mutations in the runt-related transcription factor 2 (*RUNX2*) gene. As *RUNX2* is mainly expressed in osteoblasts, CCD typically affects the skeletal and dental systems. Few studies have investigated *RUNX2* mutation effects on non-skeletal systems. Here, we describe limb-girdle myopathy, an uncommon phenotype of CCD, in a patient with a heterozygous missense mutation (p.R225Q) in the *RUNX2* gene.

**Case presentation:**

A 58 year-old man presented with progressive back pain and six months of weakness in the proximal parts of all four limbs. Physical examinations showed that he was short in stature (height, 164.4 cm; weight, 79.1 kg) with a dysmorphic face, including hypertelorism, midface hypoplasia, and chin protrusion. At a young age, he had received orthodontic surgery, due to dental abnormalities. Neurological examinations revealed sloping shoulders, weakness, and atrophy in the proximal areas of the arms, shoulder girdle muscles, and legs. The deep tendon reflex and sensory system were normal. Radiological examinations revealed mild scoliosis, shortened clavicles, and a depressed skull bone, which were consistent with a clinical diagnosis of CCD. Electromyography (EMG) studies showed myogenic polyphasic waves in the deltoid, biceps brachii, and rectus femoris muscles. Instead, the EMG findings were normal in the first dorsal interosseous, tibialis anterior and facial muscles. The EMG findings were compatible with a limb-girdle pattern with facial sparing. The patient’s family history showed his father and eldest daughter with similar dysmorphic faces, skeletal disorders and proximal upper extremity weakness. We sequenced the *RUNX2* gene and discovered a heterozygous missense mutation (c.G674A, p.R225Q), which altered the C-terminal end of the RUNX2 protein. This mutation was predicted to inactivate the protein and might affect its interactions with other proteins. This mutation co-segregated with the disease phenotypes in the family.

**Conclusions:**

We described limb-girdle myopathy in a patient with CCD that carried a heterozygous *RUNX2* missense mutation. This uncommon phenotype expanded the phenotypic spectrum of the *RUNX2* p.R225Q mutation. The role of *RUNX2* in myogenic development merits future studies. Our findings remind clinicians that myopathic patients with myopathies combined with facial dysmorphism and shortened clavicles should consider the diagnosis of CCD.

## Background

Cleidocranial dysplasia (CCD, OMIM 119600) is a rare skeletal disorder of autosomal dominant inheritance with an estimated incidence of 1/1,000,000 [1]. The main clinical features of CCD include persistent, open cranial sutures with bulging calvaria; hypoplasia or aplasia of the clavicles; dental anomalies; a short middle phalanx in the fifth finger; and associated vertebral anomalies [2]. This condition is primarily caused by mutations in the gene that encodes runt-related transcription factor 2 (RUNX2, MIM #600211), located on chromosome 6p21 [3]. The *RUNX2* gene encodes a 521 amino-acid protein with a highly conserved region of 128 amino acids (runt domain), an N-terminal stretch of glutamine/alanine repeats (Q/A domain), and a C-terminal proline/serine/threonine-rich (PST) domain [2]. The RUNX2 protein functions mainly in osteoblast differentiation and regulation [4], which contributes to the skeletal and dental abnormalities observed in CCD. In addition to its expression in osteoblasts, the RUNX2 protein is also present in non-osteoblast cells, including skeletal and smooth muscle cells [5], T cells [6], sperm cells, and neurons [7]. However, few patients with CCD manifest extra-skeletal manifestations. Here, we describe limb-girdle myopathy, an uncommon phenotype in CCD, in a patient with a heterozygous missense mutation in the *RUNX2* gene.

## Case presentation

**Fig. 1:**
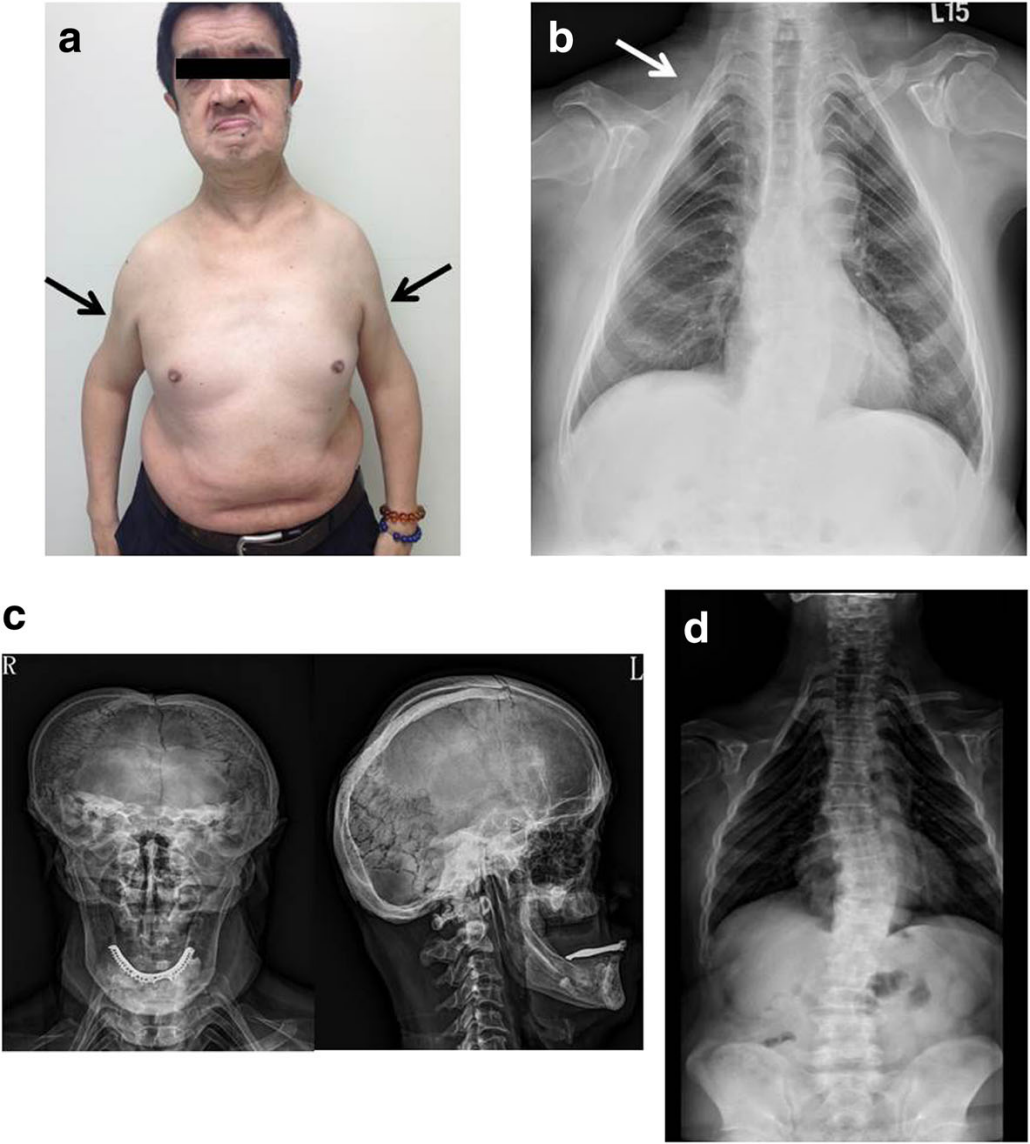
Clinical pictures and radiologic findings for the index patient with CCD. **a** The photographs show a flat face, sunken nasal bridge, hypertelorism, chin protrusion, and sloping shoulders. Arrows indicate atrophy of bilateral shoulder girdle muscles. **b** Chest X-ray shows the absent right clavicle (arrow) and a hypoplastic left clavicle. **c** Skull X-ray shows wormian bones in the skull (left panel) and a hypoplastic maxilla (right panel). **d** Spine X-ray reveals mild scoliosis of the thoraco-lumbar spine

**Fig. 2:**
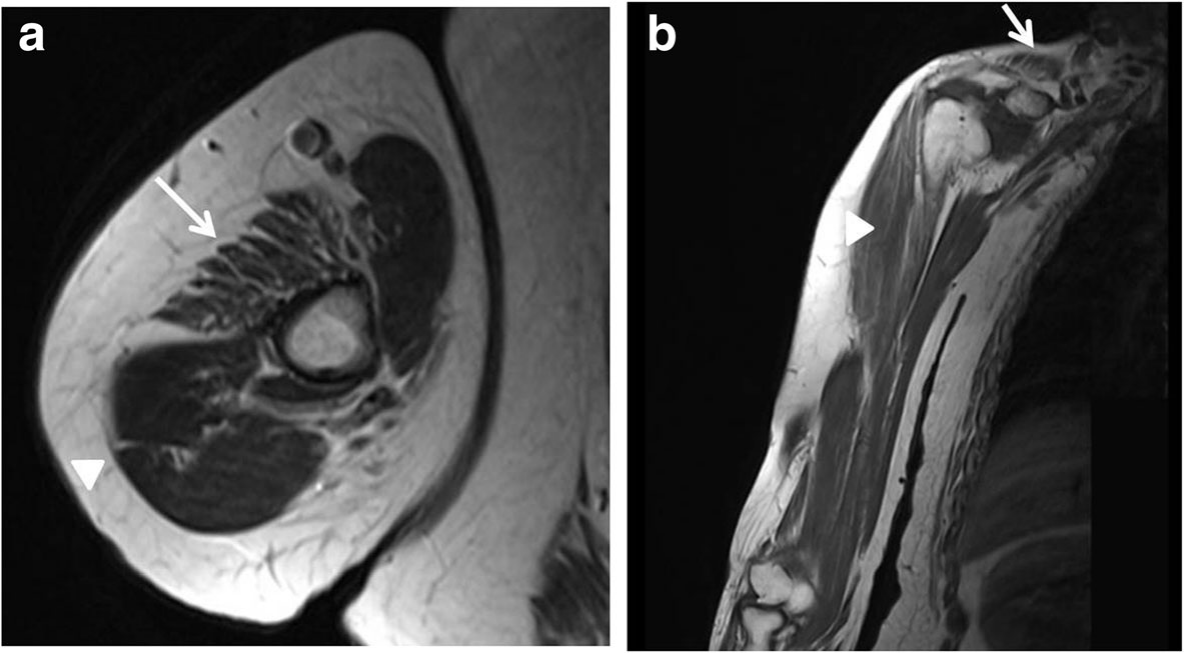
MRI of right upper extremity of the index patient. **a** Coronal T1 weighted MRI shows atrophy and fatty infiltration of the deltoid (arrow), and biceps brachii (arrowhead) muscles. **b** Sagittal T1 weighted MRI displays concomitant atrophy and fatty infiltration of the trapezius (arrows) and biceps brachii (arrowhead) muscles

**Fig. 3:**
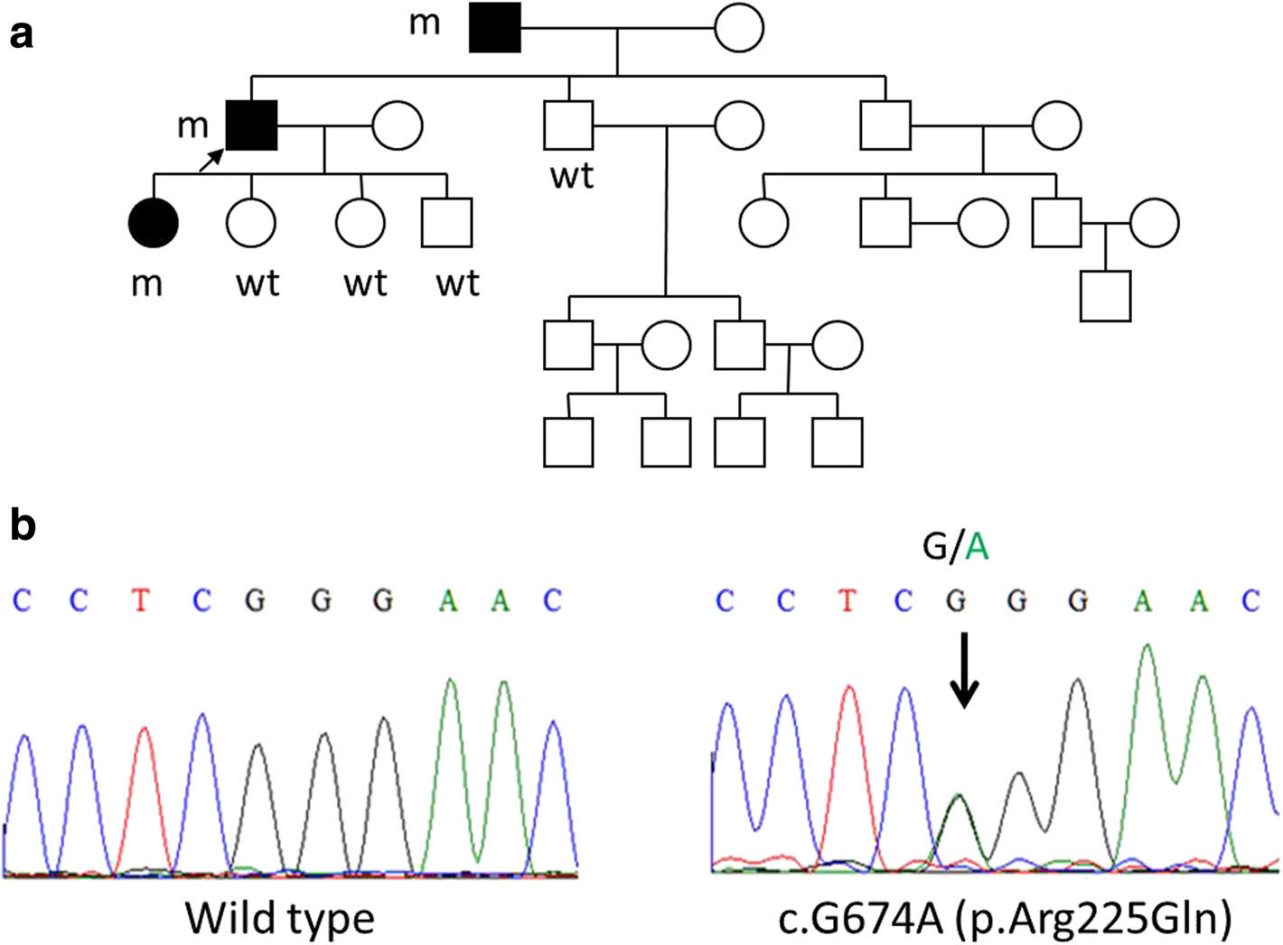
Family pedigree and genetic analysis of the *RUNX2* gene of the index patient with CCD. **a** Family pedigree of the index family. Black symbols denote family members affected with CCD. The proband we described in the current study is marked with an arrow. m, mutated alleles; wt, normal alleles. **b** Chromatograms of direct sequencing of the *RUNX2* genomic sequence. Genetic analysis reveals a single nucleotide change (c.674G > A, p.Arg225Gln, right panel) compared to the wild type sequence (left panel). The mutations identified in this study were located in the indicated position

We examined a 58-year-old man with no underlying medical conditions. He presented with progressive back pain and 6 months of weakness in the proximal parts of all four limbs. There was no diplopia, chocking, swallowing difficulty, drop neck, respiratory distress or other symptoms of heart failure while tracing back his history. Physical examinations showed he was short in stature, with a height of 164.4 cm and a weight of 79.1 kg. He had a dysmorphic face with hypertelorism, midface hypoplasia, and chin protrusion (Fig. 1a). He had received orthodontic surgery due to dental abnormalities while he was young. Neurological examinations revealed sloping shoulders, atrophy of the shoulder girdle muscles, and symmetrical weakness in the proximal parts of the arms and legs. There was no facial weakness or limitation of extra-ocular movement. The deep tendon reflex and sensory systems were normal. Radiological examinations revealed wormian bones in the skull, the absence of the right clavicle, hypoplasia of the left clavicle, and mild scoliosis. These signs were consistent with the clinical diagnosis of CCD (Fig. 1b–d). Electromyography (EMG) revealed myogenic polyphasic waves of small amplitude and short-duration polyphasic waves at 400–500 μV in the bilateral biceps brachii, deltoid, infraspinatus, trapezius, and rectus femoris muscles. However, the EMG findings were normal in bilateral first dorsal interosseous, tibialis anterior and facial muscles. Magnetic resonance imaging (MRI) of the upper extremity showed atrophy associated with fatty infiltration of right deltoid and biceps brachii muscles without acute edematous changes, suggesting a chronic muscle insult (Fig. 2a, b). These findings indicated a limb-girdle distribution of chronic myopathy. Laboratory data, including the serum creatine kinase level, were normal. The thyroid function, tumor markers, and autoimmune profiles for survey of common metabolic and inflammatory myopathy were within normal limits. Muscle biopsy of right biceps brachii muscle was suggested but this suggestion was declined by the patient. The patient did not have symptoms of respiratory distress or heart failure sign. Cardiac echo showed normal cardiac contractility and the heart ejection fraction is within normal limits. The patient’s family history showed that his father and eldest daughter had similar skeletal, dental disorders and proximal weakness of upper extremity (Fig. 3a). EMG findings of bilateral deltoid, biceps brachii and rectus femoris muscles of the proband’s elder daughter also showed myopathic polyphasic waves. These observations suggest that the myopathy is not a sporadic condition associate with the patient but segregate with the skeletal problems within his family.

The patient initially presented with facial dysmorphism, sloping shoulders, atrophy, and weakness, predominantly over the shoulder girdle. Therefore, we analyzed the double homeobox *DUX4* gene for facioscapulohumeral muscular dystrophy. In addition, the radiologic findings and the patient’s autosomal-dominant family history were consistent with CCD. Therefore, we also analyzed the *RUNX2* gene after the patient provided informed consent. Genomic DNA was extracted from peripheral whole blood for PCR analysis. We amplified exons 1 through 8 and the flanking intronic sequences of the *RUNX*2 gene with eight pairs of PCR primers, designed as previously described [8]. Genetic analysis for the *DUX4* gene was performed as previously described [9]. The purified PCR products were sequenced in both directions at our on-site biochemistry sequencing facility with Big Dye Version 3.1 and a 3730 XL sequencer (Applied Biosystems, Foster City, CA). We found that the patient carried a heterozygous missense mutation (c.G674A, p.R225Q), located at the C-terminal end of the RUNX2 protein. This mutation was predicted to inactivate the protein, and it might also affect protein structural stability and interactions with other proteins. The reference sequence and base-pair numbers refer to GenBank accession numbers AF001443-AF001450 and NM_001024630. This mutation co-segregated with the disease phenotype in the patient’s family (Fig. 3B). Research protocol was reviewed by the institutional ethics board committee of the National Taiwan University Hospital and all subjects gave informed consent.

## Conclusions

To date, variable *RUNX2* mutations have been described in subjects with CCD, including insertions, deletions, nonsense, and missense mutations. CCD has been reported in people of Mongoloid ethnicity, including Japanese, Korean, Chinese, and Taiwanese [10–13]. In most cases, patients present with classical skeletal and dental abnormalities that appear in childhood. Here, we described a patient with CCD that exhibited an uncommon initial manifestation of late-onset, limb-girdle myopathy, due to a missense mutation (c.G674A, p.R225Q) in the *RUNX2* gene.

The missense mutation, p.R225Q, located at the C-terminal end of the runt domain, is a known mutational hotspot in the *RUNX2* gene [14, 15]. The runt domain is a highly conserved DNA-binding domain, which binds to a consensus DNA sequence. The runt domain also mediates binding to CBFβ, an unrelated partner protein that does not interact directly with DNA, but enhances the DNA-binding affinity of the RUNX protein [16]. In our patient, the substitution of Arg225 with glutamine abolished the positive charge of the protein at this position, which was predicted to impair the DNA binding ability of RUNX2 [15]. Therefore, the p.R225Q mutation can cause severe phenotypes in CCD.

The p.R225Q mutation was previously reported in a Taiwanese patient with sporadic CCD. The age of onset was 8 years old, and the patient presented with classical bone phenotypes that had appeared in childhood [10]. This mutation was later reported in a teenage Korean patient with sporadic CCD, who also presented with dental and skeletal problems [11]. Those carriers of the *RUNX2* p.R225Q mutation reported no neurological complaints; in particular, there were no symptoms or signs of myopathy. In addition, a nearby nucleotide missense mutation, which changed the same amino acid, c.C673T (p.R225W), was previously described in one Italian family, but no atypical features were reported [17]. Those observations reinforced the notion that Arg225 played a vital role in the function of the RUNX2 protein, and the mutation carriers most commonly exhibited childhood or juvenile onset of dental or skeletal abnormalities, without neurological system involvement.

Notably, the patient in the present study initially presented with an upper extremity, predominant limb-girdle myopathy. As extra-skeletal presentation, especially myopathy, is not common in patients with CCD, we performed computerized English language literature searches to identify case reports of myopathy or other related neurological presentations in patients with CCD, making use of the following databases: PubMed (from 1975), and Ovid MEDLINE (from 1975). We used the following main key words for searches: cleidocranial dysplasia, myopathy, weakness, atrophy and neurological symptoms. For each selected article, the clinical descriptions of study subjects were carefully reviewed. After carefully reviewing more than 500 case studies, 13 studies reported their CCD patients present with muscle weakness. Among these 15 reported cases, 2 were found to carry *FIG 4* gene mutation without *RUNX2* gene mutations, 1 patient was diagnosed as Ritscher-Schinzel syndrome, 2 subjects’ weakness were secondary to cervical myelopathy, 8 cases were lacking of detailed descriptions of the weakness and the remaining 2 cases presented with limb-girdle myopathy (Table 1). In those cases, CCD was related to the c.G389A nonsense mutation (p.W130X) and a mutation in the exon 2-intron junction splice site (IVS2 + 2 T > A) [17]. The p.W130X mutation produced a protein predicted to lack 390 amino acids, including part of the runt and PST domains. The subject with the IVS2 + 2 T > A mutation exhibited mild conductive deafness, in addition to myopathy. Histological and histochemical analyses of a muscle biopsy specimen from the patient that carried the p.W130X mutation revealed minor, non-specific, myopathic changes [17]. These observations, combined with our findings, suggested that muscular weakness was not associated with mutations in any particular protein domain; thus, muscle weakness might be related to the loss of function of the gene product. Recent studies have shown that the expression of the *RUNX2* transcription factor may not be limited to osteoblasts and mesenchymal stem cells; expression was also found in non-osteoblast cells [5–7]. Several lines of evidence have indicated that RUNX2 also plays an important role in adipogenesis and skeletal muscle differentiation [18, 19]. RUNX2 stimulated the trans-differentiation of primary skeletal myoblasts into a mineralizing osteoblastic phenotype. RUNX2 also prevented myogenesis and myotube formation via the suppression of MyoD and myogenin transcripts [19]. Thus, RUNX2 broadly modulates cellular fates, including the fate of skeletal muscle cells. Therefore, we hypothesized that mutations in the *RUNX2* gene may lead to damaging effects in muscle cell maturation, which result in myopathy.

**Table 1:** Clinical and genetic findings in three CCD patents presented with myopathy

Subjects/reference	Classical CCD	Involvement of weakness	Other neurological signs	EMG findings	MRI findings	Muscle biopsy	*RUNX2* mutation
Case 7, Tessa et al., 2003 [17]	+	Limb-girdle myopathy	Conductive deafness	N.A.	N.A.	N.A.	Exon 2-intron junction (IVS2 + 2 T > A)
Case 4, Tessa et al., 2003 [17]	+	Limb-girdle myopathy	-	N.A.	N.A.	N.A.	c.389G > A (W130X)
Index patient of the current study	+	Limb-girdle myopathy	-	Short-duration, small-amplitude polyphasic waves	Atrophy with fatty infiltration patterns	N.A.	c.674G > A (R255Q)

*CCD* cleidocranial dysplasia, *EMG* electromyography, *MRI* magnetic resonance imaging, *N.A*. not available

In summary, this case study described an uncommon phenotype of late-onset limb-girdle distribution myopathy in a patient with CCD, associated with a *RUNX2* p.Arg225Gln mutation. Considering the role of the *RUNX2* transcription factor in myogenesis, it is not surprising that a carrier of the *RUNX2* mutation presented with extra-skeletal features. Our findings extended the phenotypic spectrum of the *RUNX2* p.R225Q mutation, from typical dental/skeletal abnormalities to a limb-girdle pattern of myopathy. Our results also remind clinicians that patients with myopathies that present with facial dysmorphism and dental or skeletal deformities should be differentially diagnosed for CCD.

## Abbreviations

CCD: Cleidocranial dysplasia
RUNX2: Runt-related transcription factor 2
